# Over 30 Years of Pediatric Liver Transplantation at the Charité—Universitätsmedizin Berlin

**DOI:** 10.3390/jcm11040900

**Published:** 2022-02-09

**Authors:** Simon Moosburner, Leke Wiering, Safak Gül-Klein, Paul Ritschl, Tomasz Dziodzio, Nathanael Raschzok, Christian Witzel, Alexander Gratopp, Stephan Henning, Philip Bufler, Moritz Schmelzle, Georg Lurje, Wenzel Schöning, Johann Pratschke, Brigitta Globke, Robert Öllinger

**Affiliations:** 1 Department of Surgery, Campus Charité Mitte and Campus Virchow-Klinikum, Charité—Universitätsmedizin Berlin, Corporate Member of Freie Universität Berlin, Humboldt-Universität zu Berlin and Berlin Institute of Health, 10117 Berlin, Germany; safak.guel@charite.de (S.G.-K.); Paul.Ritschl@charite.de (P.R.); tomasz.dziodzio@charite.de (T.D.); nathanael.raschzok@charite.de (N.R.); christian.witzel@charite.de (C.W.); moritz.schmelzle@charite.de (M.S.); georg.lurje@charite.de (G.L.); wenzel.schoening@charite.de (W.S.); johann.pratschke@charite.de (J.P.); brigitta.globke@charite.de (B.G.); robert.oellinger@charite.de (R.Ö.); 2 BIH Charité Clinician Scientist Program, Berlin Institute of Health (BIH), 10178 Berlin, Germany; 3 Department of Gastroenterology, Charité—Universitätsmedizin Berlin, Corporate Member of Freie Universität Berlin, Humboldt-Universität zu Berlin and Berlin Institute of Health, 10117 Berlin, Germany; leke.wiering@charite.de; 4 Department of Pediatrics, Section Intensive Care and Emergency Medicine, Campus Virchow-Klinikum, Charité—Universitätsmedizin Berlin, Corporate Member of Freie Universität Berlin, Humboldt-Universität zu Berlin and Berlin Institute of Health, 10117 Berlin, Germany; alexander.gratopp@charite.de; 5 Department of Pediatrics, Division of Gastroenterology, Campus Virchow-Klinikum, Charité—Universitätsmedizin Berlin, Corporate Member of Freie Universität Berlin, Humboldt-Universität zu Berlin and Berlin Institute of Health, 10117 Berlin, Germany; stephan.henning@charite.de (S.H.); philip.bufler@charite.de (P.B.)

**Keywords:** pediatric liver transplantation, survival, early allograft dysfunction

## Abstract

Background: Pediatric liver transplantation (LT) is the treatment of choice for children with end-stage liver disease and in certain cases of hepatic malignancies. Due to low case numbers, a technically demanding procedure, the need for highly specialized perioperative intensive care, and immunological, as well as infectious, challenges, the highest level of interdisciplinary cooperation is required. The aim of our study was to analyze short- and long-term outcomes of pediatric LT in our center. Methods: We conducted a retrospective single-center analysis of all liver transplantations in pediatric patients (≤16 years) performed at the Department of Surgery, Charité – Universitätsmedizin Berlin between 1991 and 2021. Three historic cohorts (1991–2004, 2005–2014 and 2015–2021) were defined. Graft- and patient survival, as well as perioperative parameters were analyzed. The study was approved by the institutional ethics board. Results: Over the course of the 30-year study period, 212 pediatric LTs were performed at our center. The median patient age was 2 years (IQR 11 years). Gender was equally distributed (52% female patients). The main indications for liver transplantation were biliary atresia (34%), acute hepatic necrosis (27%) and metabolic diseases (13%). The rate of living donor LT was 25%. The median cold ischemia time for donation after brain death (DBD) LT was 9 h and 33 min (IQR 3 h and 46 min). The overall donor age was 15 years for DBD donors and 32 years for living donors. Overall, respective 1, 5, 10 and 30-year patient and graft survivals were 86%, 82%, 78% and 65%, and 78%, 74%, 69% and 55%. One-year patient survival was 85%, 84% and 93% in the first, second and third cohort, respectively (*p* = 0.14). The overall re-transplantation rate was 12% (*n* = 26), with 5 patients (2%) requiring re-transplantation within the first 30 days. Conclusion: The excellent long-term survival over 30 years showcases the effectiveness of liver transplantation in pediatric patients. Despite a decrease in DBD organ donation, patient survival improved, attributed, besides refinements in surgical technique, mainly to improved interdisciplinary collaboration and management of perioperative complications.

## 1. Introduction

For pediatric patients with end-stage liver disease and certain hepatic malignancies, liver transplantation remains the treatment of choice [1,2]. Since the first successful pediatric liver transplantation (LT) in a patient with hepatoblastoma by Thomas Starzl in 1967, the procedure has undergone radical changes [3,4,5]. Previously reported 1-year survival rates of 11–39% are now up to 90% and children under one year of age make up almost one third of transplanted patients [6]. Indications, immunosuppression, and operative techniques have significantly changed. Surgical techniques for pediatric liver transplantation include full size orthotopic liver replacement, cadaveric split liver transplantation reduced size liver transplantation and, in rare cases, auxiliary liver transplantation, as well as living donor liver transplantation (LDLT) [7,8,9,10]. The latter, even though technically challenging, has improved the treatment of children in need of liver transplantation dramatically as many organs from cadaveric donors, in an ageing donor population, are not eligible for pediatric liver transplantation. As such, predictors of outcome and strategies to optimize patient management need to be carefully evaluated [11,12]. 

In pediatric liver transplantation, different surgical approaches, inevitable size mismatches with the donor, anatomical variants and previous abdominal surgeries can be a challenge for the entire interdisciplinary team. Despite readily available data on survival and outcome, detailed information on long term graft function, re-transplantation rates and development of interdisciplinary care are still lacking. After the first successful liver transplantation in our transplant program in 1988, and more than three thousand liver transplantations later, we decided to critically analyze the short- and long-term outcomes of our pediatric liver transplant cohort. 

## 2. Materials and Methods

### 2.1. Study Design

All pediatric liver transplantations conducted at the Charité—Universitätsmedizin Berlin between 1991 and 2021 were included into this retrospective single center cohort study. Pediatric LT patients were defined according to the Eurotransplant Liver Allocation System, as being 16 years of age or younger. Data were collected from our transplantation database and the electronic patient records that were implemented in 2005. Thus, based on data availability as well as procedural and staff changes, we defined three historic cohorts (1991–2004, 2005–2014 and 2015–2021). As previously reported [13], vascular anastomoses for especially small artery diameters were routinely carried out under a surgical microscope by a plastic surgeon, and the interdisciplinary collaboration between pediatric hepatologists, intensive care unit specialists and transplant surgeons was intensified and formalized as of 2015. 

Primary endpoint of this study was the overall patient and graft survival. Secondary endpoints were the rates of early allograft dysfunction and re-transplantation rates. Model for end-stage liver disease (MELD) and pediatric end-stage liver disease (PELD) scores were calculated as preoperative allocation parameters [14,15]. MELD was used for patients 12 years and older and PELD for patients younger than 12 years. Data on the type of graft and donation, donor and recipient age, sex, length of hospital stay, length of intensive unit care stay, last donor laboratory values before donation, recipient and graft survival, as well as on postoperative laboratory values (i.e., alanine-aminotransferase (ALT), aspartate-aminotransferase (AST), international normalized ration (INR), and bilirubin) were collected. Early Allograft Dysfunction (EAD) was calculated, as defined by Olthoff et al. [16], as bilirubin ≥ 10 mg/dL on day 7, INR ≥ 1.6 on day 7, and ALT or AST > 2000 U/L during the first 7 days. Graft loss was defined as the need for re-transplantation or recipient death due to graft failure. Patients were followed-up in our specialized pediatric LT outpatient clinic and transferred to the adult LT outpatient clinic on their 18th birthday. Routine follow-up visits follow a standardized protocol. The study was approved by the institutional ethics board of the Charité—Universitätsmedizin Berlin (EA1/035/21).

### 2.2. Surgical Technique and Postoperative Care

Liver transplantation technique depended on the type of donor, graft, and recipient. Grafts from donation after brain death (DBD) were accepted after allocation by an interdisciplinary team of pediatricians and surgeons depending on assumed graft quality according to donor history, laboratory data, imaging and, in some cases, pathology. LDLT donors were carefully evaluated and seen by the ethics board of the German federal medical association. LDLT donors underwent open or laparoscopic resection. In high-urgency cases, living donors were evaluated, but in case of a suitable, timely organ offer, transplantation was carried out with an organ from a DBD donor. LDLT grafts and cadaveric donor split grafts were transplanted in a modified piggy-back technique, using the recipient’s unified left and middle (and in very small recipients, right) hepatic vein for venous anastomosis. Full size grafts were transplanted with caval replacement and end-to-end anastomosis for the portal vein and the hepatic artery. Since 2015, hepatic artery anastomosis was carried out as an end-to-end anastomosis with a surgical microscope using interrupted 9–0 nylon sutures. Bile duct anastomosis was either carried out in an end-to-end fashion or as a hepaticojejunostomy with Roux-en-Y loop. Patients were routinely monitored on the pediatric intensive care unit after transplantation. Postoperative immunosuppression varied due to the long study period. The most recent standard of care includes a calcineurin inhibitor (tacrolimus with a targeted trough level of 8–10 ng/dL within the first 4 weeks), corticosteroids and, when indicated, mycophenolate mofetil. Induction therapy was used in patients with a high immunological risk (basiliximab, anti-thymocyte globulin or alemtuzumab).

### 2.3. Missing Data

As this retrospective analysis covers a broad timespan with several different analogue and electronic documentation systems for clinical data, most laboratory values and, consequently, classification of MELD, PELD and EAD are only available for patients transplanted after the year 2004 (group “2005–2014” and group “2015–2021”). Lost to follow-up was defined as more than five years after last visitation. Missing data were excluded from analysis. 

### 2.4. Statistical Analysis

Statistical analysis was performed with R version 4.0.3 and R Studio version 1.4 for macOS (R Foundation for Statistcal Computing, Vienna, Austria) [17]. Additional required packages were tidyverse, survival, survminer and gtsummary. For descriptive analysis, data were analyzed using the Kruskal–Wallis test. Data were reported as median and interquartile range (IQR). Categorical values were compared using the Pearson’s chi-squared or Fisher´s exact test and are reported as counts and proportions. Graft and patient survival were analyzed using Kaplan–Meier curves and compared using the log-rank test. Overall, two-sided *p* values ≤ 0.05 were considered statistically significant. 

## 3. Results

### 3.1. Data Completeness

The median follow up was 7.3 years after transplantation (IQR 14.7 years). A total of 37 patients were lost to follow-up (19.8% overall; 24.1% 1991–2004; 24.6% 2005–2014; 0% 2015–2021). Preoperative MELD or PELD values, as well as postoperative laboratory parameters and length of hospital stay were available for all patients transplanted after May 2004 (*n* = 117, 55.2%). Complete donor data were available after 2000 (*n* = 156, 73.6%).

### 3.2. Recipient Characteristics

**Figure 1:**
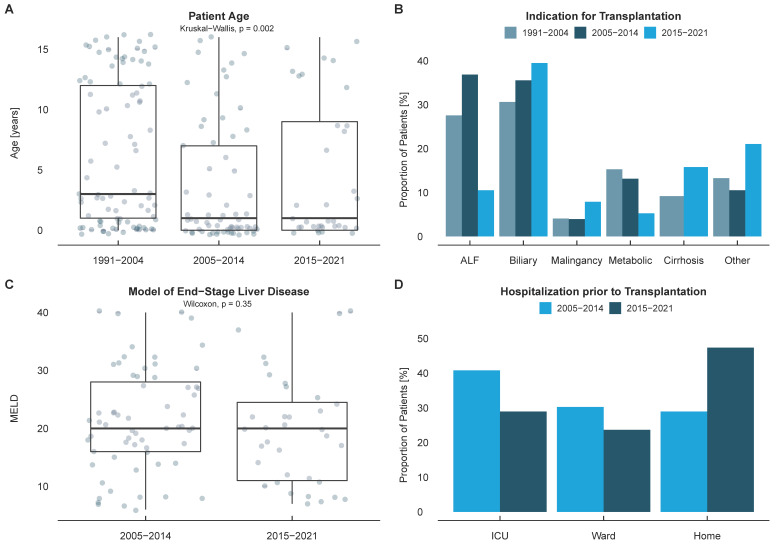
Univariate analysis of patient data. (**A**) Patient age over the three historic cohorts. (**B**) Indication of pediatric liver transplantation. (**C**) Laboratory Model of End-Stage Liver Disease (MELD) (**D**) Hospitalization prior to transplantation.

Over the course of the 30-year study period, 212 pediatric liver transplantations were performed in 187 patients at the Charité—Universitätsmedizin Berlin. Gender was equally distributed (52% female patients) and the median patient age was 2 years (IQR 11 years). The main indication for liver transplantation was biliary atresia (34%), followed by acute liver failure (27%) and metabolic diseases (13%). The median labMELD, as a surrogate parameter of the severity of the liver disease before transplantation was 20 points (IQR 18). For patients younger than 12 years, the median PELD was 18.1 (IQR 20.5). Twenty-nine percent of patients were transplanted as high urgency candidates. Thirty-one percent of high urgency candidates were patients listed for re-transplantation. Patients were hospitalized prior to transplantation in 61% of cases. Pre-transplant hospitalization was equally distributed between the ICU and the general ward (Figure 1, Table 1).

### 3.3. Donor Characteristics

The overall rate of pediatric LDLT was 25% (1991–2004: 12%; 2005–2014: 38%; 2015–2021: 32%; *p* < 0.001) (Table 2). Fifty-one percent of all DBD grafts were partial grafts. The median cold ischemia time for donation after brain death (DBD) LT was 9 h and 33 min (IQR 3 h and 46 min). The median cold ischemia for LDLT was 58 min (IQR 35 min) and decreased over time (1991–2004: 88 min; 2005–2014: 57 min; 2015–2021: 38 min; *p* = 0.003). Donor sex was equally distributed. The median overall donor age was 26 years (IQR 25 years). There were no differences in donor age over the different cohorts. Transaminase levels of DBD donors were in a normal range (median AST 60 U/L IQR 100 U/L; ALT 32 U/L IQR 54 U/L) and did not differ in between time periods (*p* = 0.6). This equally applied for donor bilirubin (6.8 µmol/L IQR 5.4 µmol/L; *p* = 0.3) and sodium (142 mmol/L IQR 13 mmol/L; *p* = 0.2). The median ICU stay before donation of DBD donors significantly increased from 2 days (IQR 2.6 days) from 1991–2004, until 4 days (IQR 5.4 days) from 2015–2021 (*p* = 0.01).

### 3.4. Patient Survival

**Figure 2:**
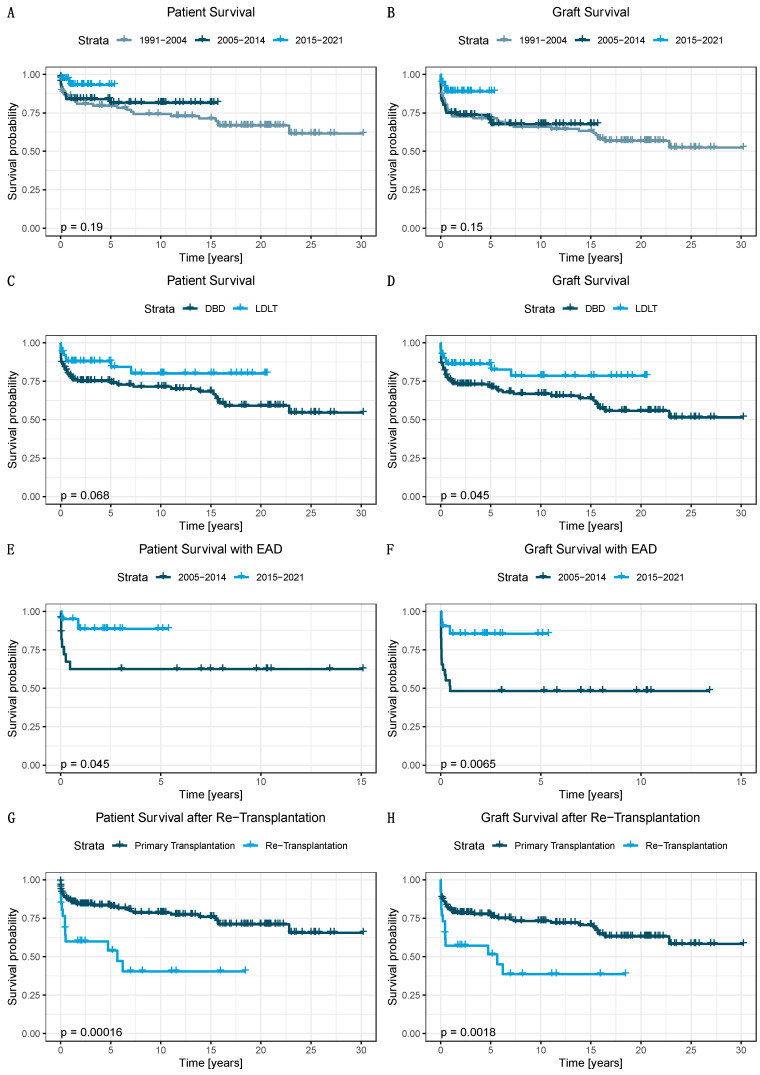
Patient and graft survival after pediatric liver transplantation. *p*-value: log-rank comparison of survival curves. (**A**) Patient survival compared over three cohorts. (**B**) Graft survival compared over three cohorts. (**C**) Patient survival in Living Donor Liver Transplantation and Donation after Brain Death Transplantation. (**D**) Graft survival in Living Donor Liver Transplantation and Donation after Brain Death Transplantation. (**E**) Patient survival in patients with early allograft dysfunction in the last two cohorts. (**F**) Graft survival in patients with early allograft dysfunction in the last two cohorts. (**G**) Patient survival after re-transplantation. (**H**) Graft survival after retransplantation. Abbreviations: Early Allograft Dysfunction (EAD), Donation after Brain Death (DBD), Living Donor Liver Transplantation (LDLT).

Overall, 1-, 5-, 10- and 30-year patient survival was 86%, 82%, 78% and 65%, respectively. Comparing the different time periods (1991–2004, 2005–2014 and 2015–2021), 1-year patient survival rates were 85.8%, 84% and 93.3%, and 5-year patient survival rates were 79.7, 81.6% and 93.3%, respectively (log-rank *p* = 0.19, 1991–2004 vs. 2015–2021 *p* = 0.11, Figure 2A). 

### 3.5. Graft Survival

Overall 1-, 5-, 10- and 30-year (non-censored by death) graft survival was 78%, 74%, 69% and 55%. Comparing the different historic cohorts (1991–2004, 2005–2014 and 2015–2021), 1-year graft survival rates were 75.9%, 75% and 88.9%, and 5-year graft survival rates were 71.6%, 69.7% and 88.9%, respectively (log-rank *p* = 0.15, 1991–2004 vs. 2015–2021 *p* = 0.062, Figure 2B). 

### 3.6. Donation after Brain Death and Living Donor Liver Transplantation

The percentage of -1, 5- and 10-year patient survival was 87.9%, 87.9% and 83.5% after LDLT compared to 87%, 82.1% and 78.2% in DBD donor transplantations (*p* = 0.068). The rates of 1-, 5- and 10-year graft survival for LDLT were 86.3%, 86.3% and 78.6% compared to 75.9%, 71% and 66.8% in DBD donor transplantations (*p* = 0.045). (Figure 2C,D). When evaluating the different periods (1991–2004, 2005–2014 and 2015–2021), 1-year LDLT patient survival was 91%, 86% and 92% in the respective time cohorts (*p* = 0.31). One-year graft survival for LDLT was 91%, 83%, and 92%, and 5-year graft survival 91%, 83% and 92% (*p* = 0.21). 

For DBD liver transplantation, 1-year patient survival was 87%, 83% and 94.1% after 2015, when evaluating the different periods (1991–2004, 2005–2014 and 2015–2021). Five-year patient survival was 80.2% until 2004, and then 78.9% and 94.1% in the following periods (*p* = 0.96). One-year graft survival was 76%, 75% and 88.9%, and 5-year graft survival was 72%, 69% and 88.9% (*p* = 0.75).

### 3.7. Re-Transplantation

The overall re-transplantation rate was 12% (11% re-transplantations and 1% (*n* = 2) third transplantations) with a median duration between transplantation and re-transplantation of 92 days (IQR 1218 days). The 30-day re-transplantation rate was 2% (*n* = 5), while the remaining patients (*n* = 21) were re-transplanted later. After re-transplantation 1-,5- and 10-year patient and graft survival were 59.9%, 53.9% and 40.5%, as well as 57.2%, 51.5% and 38.6%, respectively (both *p* < 0.001 vs. first transplantation). Early allograft dysfunction did not occur more frequently after re-transplantation (*p* = 0.14). In the most recent cohort, fewer patients required re-transplantation, with 5.7% compared to 12.6% (1991–2004) and 13.8% (2005–2014). After LDLT, 5.8% of patients required retransplantation compared to 13.2% after DBD LT (*p* = 0.23) (Figure 2G,H).

### 3.8. Early Allograft Dysfunction

EAD occurred in 43% of transplants over the study period, with ALT, AST and bilirubin levels normalizing in 79%, 39% and 55% of patients within the first seven postoperative days. The only identifiable univariate risk factor for the development of EAD was recipient PELD. The median PELD was 5 points higher in patients that developed EAD (*p* = 0.042). The occurrence of EAD was associated with significantly reduced patient and graft survival. Without EAD, 1-, 5- and 10-year patient survival was 96.4%, 90.5% and 90.5%, and with EAD was 75.6%, 75.6% and 75.6%. (*p* = 0.015). Without EAD, 1-, 5- and 10-year graft survival was 92.2%, 82% and 78.5%, and with EAD was 64.4%, 64.4% and 64.4% (*p* = 0.005). No risk factor for EAD could be identified in the multivariate analysis.

Patients developing EAD were retransplanted more frequently (18%, *n* = 9 vs. 6.1%, *n* = 4; *p* = 0.048). The median ICU stay of patients with EAD was 34 vs. 23 days (*p* = 0.006) and the overall hospital stay was 52 vs. 33 days (*p* = 0.029). There was a significant improvement 1-year patient survival after EAD after 2015 (88.7%), compared to 62.5% in the period of 2005–2014 (*p* = 0.045). This was equally true for graft survival (1-year survival: 85% vs. 48% *p* = 0.007) (Figure 2E,F).

## 4. Discussion

Liver transplantation as the treatment of choice for children with end stage liver disease, metabolic disorders and selected irresectable liver tumors has been established at the end of the last century and has been further developed during the last 20 years. Advances in immunosuppression and surgical techniques enable one- and five-year patient survival rates of 90% and 80%, respectively. Herein, we show the evolution of short- and long-term results of pediatric liver transplantation at the Charité—Universitätsmedizin during the last 30 years and demonstrate excellent outcomes. The cohort of recipients in this study by the means of age, diagnoses, MELD, PELD and hospitalization prior to LT was comparable to previous reported long-term studies [9,18].

Despite a considerable decrease in organ donation [11,19], patient survival increased in the last years. Organ scarcity, in general, is associated with an ageing DBD donor population [20]. However, we did not see an increase in donor age in our pediatric population. In contrast to adults, where the number of marginal organs being transplanted is increasing, in part facilitated through machine perfusion [21], the organ acceptance criteria for pediatric patients remained the same, as relevant donor parameters were similar across the different time periods. Selected cases of deceased after circulatory death (DCD) donor pediatric LTs have been reported in the literature, indicating a readiness to accept marginal grafts for pediatric patients [22]. However, as our waiting list mortality is below 5% lately (data not shown), acceptance of marginal organs, even under the conditions of organ scarcity in a country with an extremely low donation rate, does not seem to be required, due to the prioritization of children on the EUROTRANSPLANT waiting list and the option of LDLT.

The patient and graft survival rates in this study are comparable, in part superior, to those previously reported in the literature [9,23,24]. The largest series published so far on pediatric liver transplantation demonstrated 1-year survival rates of 84.4% in the last cohort (2013–2019) [25]. The Scientific Registry of Transplant Recipients’ (SRTR) data, published by Bowring et al., observed a 30-year patient survival rate of 57.5% in a cohort transplanted between 1987 and 1996, a number surpassed by our data, showing a 30-year survival of 65% [24]. More recent data from Canada show 10-year patient survival rates above 90%, which are in line with our more recent data as well as large database predictions for the next century [24,26]. These current projections for the United States claim a 30-year patient survival of up to 80% compared to the reported historic 68% (1997–2006). All these results exceed adult liver transplantation, with a 1-year patient survival of 81% [20,27,28]. 

Historically, results in pediatric LT were slightly worse when compared to adults but have drastically improved recently [29]. The reasons are technical innovation, graft selection, improved anastomosis technique, the option for LDLT, perioperative management as well as improved long-term immunosuppressive therapy. With respect to LDLT, we could additionally show better graft and patient survival in recipients of LDLT, with recent 1-year patient survival rates of 92%. This is no novelty and has been described previously; however, our results encourage the option for LDLT, if available [30,31,32].

The Olthoff criteria used for the definition of EAD have been shown to adequately predict graft survival in pediatric liver transplant recipients [33]. Indeed, in our population, 3-month graft survival was 24% lower if EAD occurred. EAD played a relevant role in the survival of almost half of our transplantations and significantly lengthened hospital stay. Only recipient PELD was an attributable recipient risk factors for the development of EAD. We could identify no donor risk factors, possibly related to stringent donor selection for all pediatric patients, or due to the limited number of cases investigated. As previously reported, a combination of donor and risk factors most likely attributes to the extreme case of ischemia-reperfusion-injury of the graft [34,35]. Prevention of early allograft dysfunction, by the means of the early diagnosis of complications, surgical and non-surgical, is therefore a key to reduce graft loss. 

The prevalence of EAD in our cohort was slightly higher, with the literature reporting between 23% and 36% for adult patients [36,37,38]. In our own center, we have observed EAD rates of up to 38% in adult patients, which, at least in part, may be attributed to organ scarcity and the extended use of marginal organs [12]. However, despite an equal EAD rate, we could show that 1- and 5-year graft survival after EAD significantly improved in the last cohort. In fact, we mainly attribute this to improved perioperative management. Reports detailing early allograft dysfunction for pediatric patients are lacking. However, we assume more complex vascular anastomosis resulting in potentially longer cold and warm ischemia times, respectively, to lead, at least in part, to higher EAD rates than in adult patients [39,40,41]. Nonetheless overall re-transplantation rates remained comparatively low, potentially indicating recuperation in selected patients. 

Our re-transplantation rate was comparable to similar single center reports [42,43] and was higher in patients with EAD and deceased donor LTs. Long term results are comparable with previously published studies [18,44]: One-year patient survival with 60% after re-transplantation was significantly worse compared to first transplants, even though there were only few early re-transplantations. Early re-transplantation, e.g., for vascular complications, itself, has been shown to have inferior survival compared to re-transplantation at a later time point [43]. 

Patient age decreased significantly across eras, which we mainly associated with a higher rate of LDLT (2015–2021: 31.6%, 2005–2014: 38.2% and 1991–2004: 12.2%). The rates of split graft transplantations from DBD donors remained the same. Diagnoses for transplants did change over time, however, with predominantly higher rates of acute liver failure before 2015. We hypothesize that improved intensive care for acute liver failure patients reduces the need for liver transplantation. 

Overall, we saw an increase in patient survival in between our historic cohorts, with the 1-year patient survival increasing by 7.5% after 2015. Similar comparisons have already been made previously [45], with Hackl et al. showing a linear increase in patient survival after the transplant year [6]. However, we show a similar patient survival between 1991 and 2015, which is the only improvement in the long-term survival comparing 2005–2014 and 1991–2004. We argue that the implementation of a new surgical approach and a strengthened interdisciplinary collaboration have attributed to this fact. For hepatic artery reconstruction, we saw great results with the reconstruction of the hepatic artery under the microscope, that led to an arterial thrombosis rate of 0%. Considering our previously published analysis, where hepatic artery thrombosis was the major risk factor for the worse outcome after pediatric LT [13], this is especially relevant. Nonetheless, overall modernization (e.g., by the means of much more sensitive ultrasound scans), improved antibiotic regimens, including antibiotic stewardship [46], and meticulous follow-up care will have impacted these results as well. Especially, the fact that the rate of EAD remained similar over the three time periods, but improvement in patient and graft survival could be achieved in the last cohort, indicates that the relevance of improvements in the early postoperative phase may be paramount. Finally, more than 30 years of experience itself have improved complication awareness for all parties involved. 

### Limitations

A significant number of patients were lost to follow up, which can be explained by the exceptionally long observational period and a significant number of patients being transplanted in our historic cohorts, who came from different countries, where pediatric LT at that time was not an option. Nowadays, in order to avoid organ tourism, regulations of Eurotransplant and the German medical association do not allow transplantations of children without a residence in Germany (except for high urgency cases with acute liver failure). Further, laboratory values from before 2005 were not available in all cases, as the medical records retention time is no longer than 10 years. Generally, this is a weakness of long-term observational studies and, especially for patients transplanted in the early days of LT, results might be inferior due to underreporting of graft failure or death.

## 5. Conclusions

Our excellent short- and long-term survival over more than 30 years of pediatric LT showcases the effectiveness of liver transplantation for pediatric patients. Despite a decrease in organ donation in recent years, patient survival improved, attributed besides refinements in surgical technique mainly to improved interdisciplinary recognition and management of perioperative complications. Our data encourage LDLT and early diagnosis and the prevention of EAD and give parents of children in need for LT an optimistic perspective. 

## Figures and Tables

**Table 1:** Patient Characteristics.

	Donation after Brain Death Liver Transplantation					Living Donor Liver Transplantation				
Variable	Overall, N = 129 ^1^	1991–2004, N = 69 ^1^	2005–2014, N = 37 ^1^	2015–2021, N = 23 ^1^	*p*-Value ^2^	Overall, N = 51 ^1^	1991–2004, N = 11 ^1^	2005–2014, N = 28 ^1^	2015–2021, N = 12 ^1^	*p*-Value ^2^
Age (years)	3 (1, 12)	7 (1, 14)	1 (0, 10)	3 (1, 12.5)	0.032	0 (0, 2)	2 (0, 9)	0 (0, 1.5)	0 (0, 0)	0.052
Sex (f)	66 (51%)	34 (49%)	20 (54%)	12 (52%)	0.9	26 (51%)	4 (36%)	14 (50%)	8 (67%)	0.3
BMI (kg/m2)	16.8 (15, 19)	16.7 (14.7, 18.2)	17.2 (15.5, 19.5)	16.1 (15.1, 18.7)	0.6	15.38 (14.20, 16.67)	15.93 (14.92, 19.42)	15.61 (14.16, 16.72)	14.96 (14.24, 15.48)	0.3
MELD	20 (11, 29)	na	22 (18, 31)	17 (11, 24)	0.08	20 (16, 24)	na	20 (16, 22)	20 (18, 26)	0.5
PELD	18 (−2, 27)	na	19 (−4, 29)	8 (0, 21)	0.3	19 (9, 27)	na	17 (8, 23)	26 (18, 32)	0.085
High Urgency	34 (41%)	7 (32%)	18 (49%)	9 (39%)	0.4	5 (9.8%)	na	3 (11%)	2 (17%)	0.4
LOS (d)	40 (28, 71)	60 (44, 76)	36 (27, 54)	42 (28, 84)	0.5	34 (26, 68)	na	31 (26, 62)	47 (29, 75)	0.5
ICU (d)	25 (15, 38)	na	23 (14, 35)	31 (16, 57)	0.3	26 (21, 46)	na	26 (21, 49)	26 (21, 39)	0.8

^1^ Median (IQR); n (%) ^2^ Kruskal–Wallis rank sum test; Pearson’s Chi-squared test; Fisher’s exact test. Abbreviations: Body Mass Index (BMI), Model for End-Stage Liver Disease (MELD), Pediatric End-Stage Liver Disease (PELD), High Urgency (HU), Length of Hospital Stay (LOS), Intensive Care Unit (ICU).

**Table 2:** Donor Characteristics.

	Donation after Brain Death Liver Transplantation					Living Donor Liver Transplantation				
Variable	Overall, N = 152 ^1^	1991–2004, N = 79 ^1^	2005–2014, N = 47 ^1^	2015–2021, N = 26 ^1^	*p*-Value ^2^	Overall, N = 52 ^1^	1991–2004, N = 11 ^1^	2005–2014, N = 29 ^1^	2015–2021, N = 12 ^1^	*p*-Value ^2^
Split graft	39 (51%)	2 (67%)	20 (43%)	17 (65%)	0.2	42 (100%)	1 (100%)	29 (100%)	12 (100%)	
Age (years)	14 (8, 32)	15 (10, 31)	14 (6, 32)	14 (3, 31)	0.6	32 (28, 37)	30 (28, 36)	32 (28, 37)	33 (28, 38)	0.7
Sex (f)	46 (46%)	13 (48%)	21 (45%)	12 (46%)	>0.9	32 (62%)	6 (55%)	19 (66%)	7 (58%)	0.8
BMI (kg/m^2^)	20 (17, 22)	20 (18, 23.5)	19 (16, 22)	19.5 (17, 21)	0.4	24 (22, 25.25)	250 (22, 27)	240 (22, 25)	240 (22, 25.25)	0.8
Cold Ischemia (min)	550 (462, 661)	560 (456, 664)	523 (455, 594)	598 (527, 777)	0.013	58 (38, 73)	88 (68, 130)	57 (45, 69)	38 (14, 63)	0.003
ICU Stay (d)	2.9 (1.7, 5.9)	1.8 (0.9, 3.6)	2.4 (1.7, 5.3)	4.4 (2.4, 7.8)	0.011					
Sodium (mmol/L)	140 (137, 149)	142 (138, 153)	142 (138, 150)	140 (137, 142)	0.3					
AST (U/L)	60 (26, 130)	40 (26, 82)	64 (29, 126)	65 (23, 204)	0.5					
ALT (U/L)	32 (18, 72)	26 (18, 35)	37 (20, 66)	31 (16, 86)	0.6					
Bilirubin (µmol/L)	6.8 (5, 10.4)	8.6 (5.1, 14.9)	5.9 (4.2, 10.4)	6.6 (5, 8)	0.3					

^1^ Median (IQR); n (%) ^2^ Kruskal–Wallis rank sum test; Pearson’s Chi-squared test; Fisher’s exact test. Abbreviations: Body Mass Index (BMI), Intensive Care Unit (ICU).

## Data Availability

Data can be obtained from the corresponding author upon reasonable request.
